# Pharmacological and genetic modulation of IL-32 expression in intestinal epithelial cells does not impact HIV-1 outgrowth in co-cultured CD4^+^ T-cells

**DOI:** 10.3389/fimmu.2026.1769388

**Published:** 2026-05-26

**Authors:** Etiene Moreira Gabriel, Julie Moreaux, Chernkhwan Kaofai, Kimiya Majidi Ivari, Jean-François Schmouth, Jean-Philippe Goulet, Jonathan Dias, Tomas Raul Wiche Salinas, Laurence Raymond Marchand, Soumia Khalfi, Jean-Pierre Routy, Madeleine Durand, Mohamed El-Far, Cécile L. Tremblay, Petronela Ancuta

**Affiliations:** 1 Centre hospitalier de l'Université de Montréal (CHUM) Research Centre, Montréal, QC, Canada; 2 Department de Microbiologie, Infectiologie et Immunologie, Faculté de Médecine, Université de Montréal, Montréal, QC, Canada; 3 CellCarta, Montréal, QC, Canada; 4 McGill University Health Centre, Montreal, QC, Canada; 5 Département de Médecine, Faculté de Médecine, Université de Montréal, Montréal, QC, Canada

**Keywords:** all-trans retinoic acid, antiretroviral therapy, CD4+ T-cells, CRISPR/Cas9, HIV-1, IL-22, IL-26, IL-32

## Abstract

**Background:**

The crosstalk between intestinal epithelial cells (IEC) and CD4^+^ T-cells is essential for the maintenance of mucosal homeostasis, but its role in governing HIV-1 latency *versus* reactivation in gut-homing/resident T-cells remains poorly documented. We previously demonstrated that the Th17-lineage cytokine IL-17A transcriptionally reprograms IEC for promoting viral outgrowth in CD4^+^ T-cells of antiretroviral therapy (ART)-treated people with HIV-1 (PWH). These effects coincided with the downregulation of IL-32, a cytokine with documented antiviral properties and identified as a marker for HIV-1 disease progression and cardiovascular disease risk. Here, we aimed to identify modulators of IL-32 expression and to define the impact of IEC expressing IL-32 on HIV-1 outgrowth in neighboring CD4^+^ T-cells carrying viral reservoirs.

**Methods:**

HT-29 IEC were activated with TNF and/or IL-22, IL-26, *all*-*trans* retinoic acid (ATRA) and rosiglitazone (RGZ). CRISPR/Cas9 gene editing was used to knockout (KO) *IL32* in IEC, with efficiency/off-target effect assessments performed using TIDE and whole-genome RNA-Sequencing. IL-32β/γ/ϵ mRNA/protein were quantified by RT-PCR/ELISA. An IEC-based viral outgrowth assay (VOA) was performed with CD4^+^ T-cells of ART-treated PWH. Soluble/intracellular HIV-p24 levels were measured by ELISA/flow cytometry.

**Results:**

In combination with TNF, IL-22 upregulated IL-32β/ϵ, RGZ increased IL-32β, ATRA decreased IL-32β/γ/ϵ, while IL-26 had no impact in IEC. HIV-1 outgrowth in IEC:T-cell co-cultures was not affected by IL-22/RGZ/ATRA-mediated changes in IL-32 expression. The analysis of differentially expressed genes in control *versus IL32KO* IEC revealed minor differences in transcriptional profiles before/after exposure to TNF, while the IL-32 mRNA/protein expression was induced by TNF in control but not *IL32KO* IEC. Finally, TNF-activated control *versus IL32*KO IEC supported with similar efficacy HIV-1 outgrowth in CD4^+^ T-cells of PWH.

**Conclusions:**

We identified IL-22, ATRA and RGZ as novel regulators of IL-32 expression in TNF-primed IEC and demonstrated that pharmacological and genetic modulation of IL-32 expression in IEC has no major impact on HIV-1 outgrowth from co-cultured CD4^+^ T-cells of ART-treated PWH carrying viral reservoirs. By excluding its role in modulating HIV reservoir latency/reactivation at intestinal barrier level, these results support the idea of testing the potential use of IL-32 as a novel therapeutic target to reduce comorbidities in ART-treated PWH.

## Graphical Abstract

**Figure:**
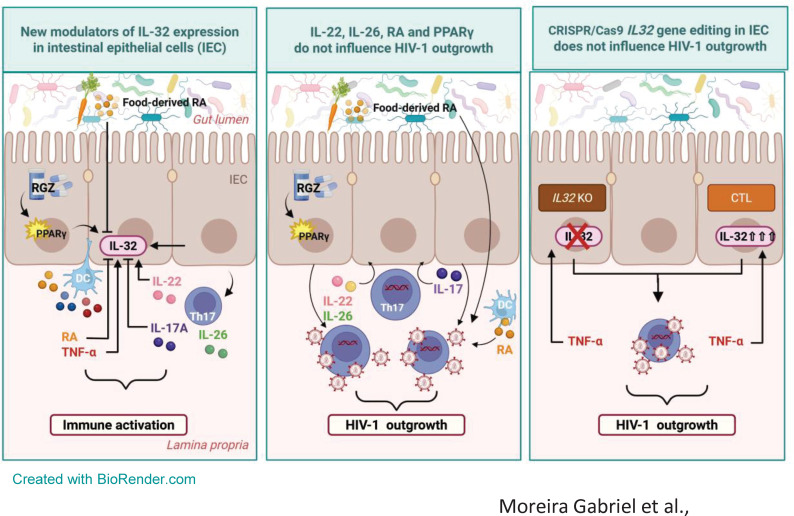
: Intestinal epithelial cells promote HIV-1 outgrowth from CD4^+^ T-cells of ART-treated PWH via mechanisms independent on IL-32. Our results identified new positive (*i.e.*, IL-22, RGZ) and negative (*i.e.*, RA) regulators of IL-32 expression in IEC that act in combination with TNF (A), demonstrated that modulators of IL-32 expression in IEC have a negligible effect on HIV-1 outgrowth (B), and that TNF-activated IEC sustain HIV-1 outgrowth in CD4^+^ T-cells of ART treated PWH *via* mechanisms independent of IL-32 expression (C).

## Introduction

The antiretroviral therapy (ART) significantly improved the quality of life of people with human immunodeficiency virus type 1 (HIV-1) (PWH) and transformed the deadly HIV-1 epidemic into a manageable chronic condition (1, 2). However, two major obstacles continue to hinder HIV-1 eradication/remission during ART: first, the persistence of viral reservoirs in long-lived immune cells that escape the sensing/clearance by the immune mechanism [*e.g.*, antibody-dependent cell cytotoxicity (ADCC), CD8^+^ T-cell-mediated killing] (1–3) and second, the incomplete restoration of mucosal barrier integrity, thus increasing the risk for comorbidities (4, 5).

Pioneering studies identified HIV-associated enteropathy as a key event in viral pathogenicity and disease progression (4). Intestinal epithelial cells (IEC) are among the first cells to encounter the virus at mucosal barrier surfaces (6–8). In addition to mechanisms involving the free virion transmission, HIV-1 is documented to bind on the epithelial receptor galactosyl ceramide (GalCer) (9–11). Captured virions are being transported across epithelial cells by transcytosis and the infection is subsequently transmitted to neighboring immune cells present in the gut-associated lymphoid tissues (GALT, *e.g.*, dendritic cells, macrophages, CD4^+^ T-cells) upon formation of immunological/virological synapses (12–17). The GALT is one major site of viral reservoir persistence during ART (18, 19). The intestinal environment is considered an active site of immune activation due to the constant interaction with the local microbiota (20, 21). However, studies reported a state of deep HIV-1 latency in colon-infiltrating CD4^+^ T-cells of ART-treated PWH (22–24), with differences in the transcriptional activity of HIV-1 reservoirs being observed between ileum, duodenum and colon (4, 5). This evidence raises new questions about factors that influence HIV-1 reservoir latency *versus* reactivation in the GALT-infiltrating CD4^+^ T-cells.

Among CD4^+^ T-cells, Th17-polarized cells contribute to the preservation of epithelial layer integrity and functions, by promoting epithelial proliferation, tissue repair, the strengthening of tight junctions, immune cell recruitment, production of antimicrobial peptides and mucins and performing immune surveillance (18, 25). Th17 cells are highly permissive to HIV-1 infection (18), represent among the earliest targets at mucosal sites of viral entry (26), and are rapidly depleted early after infection, with deficits at mucosal sites persisting even when ART was initiated during early acute infection (27). Nevertheless, a fraction of Th17 cells are long-lived and serves as HIV-1 reservoirs in both the colon and peripheral blood of ART-treated PWH (18). Th17 cells are unique in their capacity to produce lineage-specific cytokines (*i.e.*, IL-17A, IL-22, IL-26) that act on IEC to sustain mucosal barrier integrity on one side, or promote intestinal inflammation and microbial translocation on the other side (28, 29). While the crosstalk between IEC and immune cells is critical for maintaining mucosal homeostasis (20, 30–32), the role of IEC in modulating HIV-1 latency or reactivation in GALT-infiltrating CD4^+^ T-cells remains poorly investigated (5).

By using an IEC-based viral outgrowth assay (VOA), in which IEC were co-cultured with CD4^+^ T-cells of ART-treated PWH, we demonstrated that the Th17-lineage cytokine IL-17A transcriptionally reprogrammed IEC for an enhanced ability to promote HIV-1 replication/outgrowth in CD4^+^ T-cells (33). Among transcripts modulated by IL-17A in IEC, we noted a reduced expression of interferon (IFN)-stimulated genes (ISGs), including transcripts encoding for IL-32 (33), a family of cytokines involved in multiple pathologic processes (34–36) and reported to exhibit antiviral features in HIV-infected macrophages (37, 38). Our group identified IL-32 as a marker of HIV-1 disease progression (39, 40) and demonstrated overt expression of IL-32β mRNA in the colon of ART-treated PWH compared to uninfected controls (41). Moreover, we demonstrated that exposure to TNF, microbial compounds, and HIV led to increased IL-32β expression in HT-29 cells *in vitro* (41). Furthermore, we demonstrated the effect of IL-32 isoforms on T-cell functions (42, 43), including the reprogramming of CD4^+^ T-cells for a heart-homing tropism and HIV-1 permissiveness signature (40). Considering the state of deep HIV-1 latency documented in the gut of ART-treated PWH (22–24), the possibility that intrinsic IL-32 expression in IEC negatively regulates HIV-1 outgrowth in neighboring CD4^+^ T-cells carrying HIV-1 reservoirs remains one plausible hypothesis (36, 44).

To explore the antiviral role of IL-32 in the context of the crosstalk between IEC and CD4^+^ T-cells, in this study we used the IEC-based VOA to test the effect of other Th17 cytokines, IL-22 and IL-26, on IL-32 expression by IEC and its impact on HIV-1 outgrowth. In search for other new modulators of IL-32 expression, IEC were also exposed to one positive [*i.e.*, retinoic acid receptor alpha (RARA) *via* its ligand *all-trans* retinoic acid (ATRA)] and one negative [*i.e.*, peroxisome proliferator-activated receptor gamma (PPARγ) *via* its ligand rosiglitazone (RGZ)] regulator of HIV-1 permissiveness, identified by our group (45–47). We also used the CRISPR/Cas9 gene editing technology to generate *IL32 knock-out (*KO) IEC and tested the impact of IL32 silencing on HIV-1 outgrowth in CD4^+^ T-cells of ART-treated PWH. This study identified novel regulators of IL-32 expression in IEC and demonstrated that the crosstalk between IEC and CD4^+^ T-cells governs HIV-1 outgrowth *via* mechanisms independent of the IEC intrinsic IL-32 expression.

## Materials and methods

### Ethics statement

This study was conducted on peripheral blood mononuclear cells (PBMCs) of ART-treated PWH in line with the ethical principles of the Declaration of Helsinki for biomedical research involving human subjects. The study was approved by the Institutional Review Boards (IRB) of the McGill University Health Center (MUHC; Montréal, Québec, Canada; approval number MP-02-2023-11299) and the Centre de recherche du *Centre hospitalier de l’Université de Montréal* [CHUM, Montréal, Québec, Canada; approval number 2023-11299 (22.267)]. All study participants provided written informed consent (**Supplementary Table 1**) for the collection of peripheral blood through leukapheresis, the use of PBMCs for research and the publication of the results.

### Study participants

Human study participants ART-treated PWH (n=10) recruited at the McGill University Health Centre (MUHC), Montréal, Québec, Canada, with clinical parameters included in **Table 1**. PBMCs (10^9–^10^10^ cells) were collected by leukapheresis, as we previously described (33).

**Table 1:** Clinical parameters of ART-treated PWH study participants.

Participants	Sex	Ethnicity	Age	CD4 counts #	CD8 counts #	Plasma viral load&	Time since infection*	ART regimen	Time on ART*
ART #1	Male	Caucasian	21	796	399	<40	8	Stribild	4
ART #2	Male	Caucasian	49	458	899	<40	227	Truvada Viramune	201
ART #3	Male	Caucasian	51	841	1322	<40	149	Sustiva Truvada	149
ART #4	Male	Caucasian	44	398	775	<40	154	Complera	25
ART #5	Male	Caucasian	36	542	803	<40	13	Stribild	12
ART #6	Male	Latin-American	31	775	1000	<40	69	Complera	16
ART #7	Male	Caucasian	30	598	605	<40	80	Stribild	**77**
ART #8	Male	Caucasian	47	425	1156	<40	182	Atripla	60
ART #9	Male	Caucasian	59	704	836	<40	273	Symtuza	264
ART #10	Male	Caucasian	61	397	238	<40	44	Stribild	38

ART, Antiretroviral therapy; PWH, people with HIV; #, cells/µL; &, HIV-RNA copies per ml of plasma; * Months.

### HT-29 cell culture and stimulation

The HT-29 epithelial cell line isolated from a female colorectal adenocarcinoma participant (Cat. no. HTB38™, ATCC^®^, Manassas, Virginia, USA), was used as a model of IEC, as we described (33, 41). The HT-29 cells were grown in McCoy’s medium (Cat. no. 16600082, Gibco^®^, ThermoFisher Scientific, Waltham, USA), supplemented with 10% of heat-inactivated fetal bovine serum (FBS, Cat. no. 090-150-FBS, Wisent^®^, Québec, Canada) and 1% penicillin/streptomycin (P/S, Cat. no. 15140122, Gibco^®^), at 37 °C and 5% CO_2_. Cells were seeded in 25 cm^2^ and then 75 cm^2^ flasks, cultured at confluence (≅95%), and harvested using 0.05% of trypsin/EDTA (Cat. no. 25200072, Gibco^®^). Experiments were conducted with cells between passage 3 and 10, with cells being passaged every 3–6 days before reaching 100% confluence. Experiments were performed with HT-29 cells (10^5^ cells/well) plated in 48-well plates (Cat. no. 3548, Costar, Millipore Sigma) and exposed to human recombinant (hr) cytokines [*i.e.*, hrTNF-α (10 ng/mL; Cat. no. 210-TA), hrIL-17A (10 ng/mL, Cat. no. 7955-IL), hrIL-22 (50 ng/mL, Cat. no. 782-IL), hrIL-26 (50 ng/mL; Cat. no. 1870-IL), R&D Systems, Minneapolis, MN, USA)], using optimal concentrations we identified in previous studies (33, 41). Cells were also exposed to *all-trans retinoic acid* (ATRA, 50 nM; Cat. no. R2625; Millipore Sigma, St. Louis, MO, USA) and RGZ (50 µM; Cat. no. 71740, Cayman Chemicals, Ann Arbor, Michigan, USA), using optimal concentrations we identified in previous studies (45, 46). Upon 18 hours of stimulation, cytokine/drug-activated IEC were used for the IEC-based viral outgrowth assay (VOA), as described below. In parallel, a fraction of cytokine/drug-activated HT-29 cells were harvested using trypsin, washed with PBS 1X (Gibco^®^), and used for IL-32 mRNA quantification by RT-PCR, or IL-32 protein quantification by crude ELISA, or genome-wide RNA-sequencing, as described below. All investigations on HT-29 cells were conducted in triplicates, and repeated in at least three independent experiments.

### Viral outgrowth assay

The VOA was performed, as we reported (45, 46, 48). Briefly, memory CD4^+^ T-cells were enriched from PBMCs of ART-treated PWH by negative selection using magnetic beads (Cat. no. 17952, STEMCELL Technologies, Vancouver, Canada), according to the manufacturer’s instructions. Cell purity was determined upon staining with Abs against CD3 (Cat. no. 558117), CD4 (Cat. no. 612936 from BD Biosciences, San Diego, CA, USA), CD45RA (Cat. no. 47-0458-42, ThermoFisher Scientific, Waltham, USA), and CD8 (Cat. no. 130-080-601, Miltenyi, Bergisch Gladbach, Germany), followed by flow cytometry analysis (BD LSRFortessa™). Memory CD4^+^ T-cells were cultured in 48-well plates (Costar, 10^6^ cells/well), in the presence of immobilized CD3 Abs (1 μg/mL, Cat. no.555329) and soluble CD28 Abs (1 μg/mL; Cat. no. 555725, BD Pharmingen, San Diego, CA, USA) for 3 days. Activated CD4^+^ T-cells were washed and subsequently cultured in new 48-well plates in RPMI 1640 media containing 10% FBS, 1% P/S, and hrIL-2 (5 ng/ml; Cat. no. 202-IL, R&D Systems®) for up to 12 days, with 50% of the media being harvested and refreshed every 3 days. Cell-culture supernatants were used for HIV-p24 quantification by ELISA, while cells collected at day 12 post-co-culture were analyzed for productive HIV-1 infection by flow cytometry, as described below.

### IEC-based VOA

The IEC-based VOA was performed, as we reported (33). Briefly, HT-29 cells stimulated with cytokines and/or drugs for 18 hours in 48-well plates were washed with media to exclude all soluble factors. The HT-29 monolayers (≅80% confluence) were co-cultured with memory CD4^+^ T-cells of ART-treated PWH (10^6^ T-cells/well), isolated and activated as described above. IEC:T-cell co-cultures were maintained in RPMI 1640 media containing 10% FBS, 1% P/S, and hrIL-2 (5 ng/ml), for up to 12 days, with 50% media being refreshed every 3 days. Cell culture supernatants were collected for HIV-1 outgrowth quantification by ELISA and flow cytometry, as described below. All investigations were conducted in triplicates with CD4^+^ T-cells of different ART-treated PWH (**Table 1**).

### Flow cytometry identification of productively HIV-infected T-cells

Productively HIV-infected T-cells were identified in VOA and IEC-based VOA, as we reported (33, 45, 46, 48). Briefly, at day 12, cells were harvested and stained on the surface with fluorochrome-conjugated Abs against CD3 (Alexa Fluor® 700, Cat. no. 557943) and CD4 (BUV496, Cat. no. 612936, BD Biosciences, San Diego, CA, USA). The Aqua Vivid Dead Cell Stain Kit (Cat. no. L34957, ThermoFisher Scientific, Waltham, USA) was used to exclude dead cells. Intracellular HIV-p24 staining was performed using the BD Cytofix/Cytoperm kit (Cat. no. 554714, BD Biosciences, Franklin Lakes, NJ, USA) and FITC-conjugated HIV-p24 Abs (Cat. no. 6604665, Beckman Coulter, Brea, CA, USA). Phenotypic analysis was performed *via* flow cytometry using a BD LSRFortessa™ cytometer. Data acquisition and analysis were performed using BD-Diva (BD Biosciences) and FlowJo (Tree Star, Ashland, Oregon, USA) software. Productively infected cells were identified based on their CD3^+^CD4^low^HIV-p24^+^ phenotype, as depicted in **Supplementary Figure 1**.

### HIV-p24 ELISA

Cell culture supernatants containing HIV-1 virions were lysed using an in-house buffer solution [PBS 1x Wisent^®^, Tween 20 0.05%, Triton X-100 2.5%, Trypan Blue 1% and Thimerosal 0.02% (Millipore Sigma, Oakville, ON, Canada) in deionized water] for 1h at 37 °C. HIV-p24 protein levels were quantified using an in-house sandwich ELISA, as we previously described (33, 45, 46, 48).

### IL-32 β/γ/ϵ isoform RT-PCR quantification

Total RNA was extracted from HT-29 cells using the RNeasy kit (Cat. no. 74136 Qiagen, Hilden, Germany), following the manufacturer’s instructions. The IL-32β/γ/ϵ isoforms were quantified using one-step SYBR Green real-time PCR on the LightCycler 480 II (Roche, Mississauga, ON, CAN) and Qiagen reagents, as we previously reported (39, 41, 42). The experiments were performed in duplicates using 25 ng of RNA per reaction for each IL-32 mRNA isoform. Negative controls (no sample, no reverse transcriptase) were included for each set of primers. IL-32 isoform expression was normalized to the housekeeping gene β-glucuronidase (GUSB). Primers for IL-32 isoforms (β/γ/ϵ) and reference gene were used, as we previously reported (39, 41, 42). Results are presented as relative target gene quantification (ΔCT) as compared to the unstimulated condition (Media).

### Crude IL-32 ELISA

A crude IL-32 ELISA was performed on cell lysates, as we previously reported (39, 41). Briefly, HT-29 cell dry pellets were lysed by 1x RIPA buffer (Cat. no. 9806S, Cell Signaling Technology, Danvers, MA, USA) supplemented with protease inhibitors (Cat. no. 11836170001 Millipore Sigma, St. Louis, MO, USA), followed by sonication. Total protein levels in cell lysates were quantified using the DC protein assay kit (Cat, no 5000112, BioRad, Hercules, CA, USA). IL-32 protein levels were quantified using a commercial ELISA kit for total IL-32 (Cat. no. DY3040-05, R&D Systems, Minneapolis, MN, USA), according to manufacturer’s protocol. IL-32 protein levels were normalized to total protein content (pgs IL-32 protein/1 µg total protein).

### *IL32* CRISPR/Cas9 gene editing

The CRISPR-Cas9 reagents used in this study targeted the human *IL32* gene and were derived from the GeCKO V2 library (49, 50). Three custom crRNAs (IDT, Alt-R™crRNA) corresponding to specific sequences reported in **Table 2** and a generic Alt-R^®^ CRISPR-Cas9 tracrRNA were prepared at a concentration of 200 µM. A pre-genomic (pg)RNA complex was created by mixing the components at an equimolar ratio, incubating them at 95 °C for 5 minutes, and allowing them to cool to room temperature for 10 minutes. For the preparation of CRISPR-Cas9 electroporation mixes, Alt-R^®^ S.p. Cas9 HiFi Nuclease V3 (IDT) was used along with the corresponding pgRNAs. RNP complexes were formed at a 1.2:1 molar ratio in 1XPBS, and the pgRNA-Cas9 mix was incubated at room temperature for 20 minutes before electroporation. Five conditions were established: Guide 1, Guide 2, Guide 3, Pooled Guides and Negative Control. HT-29 cells were detached from the plates using Trypsin, washed, adjusted to a concentration to 1x10^6^ cells, kept on ice for at least 5 minutes and electroporated using a Gene Pulser XCell electroporator (Bio-Rad) at specific conditions (pre-cooled 2 mm cuvette, 250V, 200 µF). After electroporation, cells were allowed to recover for 24 hours before visualization via microscopy to assess the ATTO™ 550 positive signal. Finally, cells were sorted using fluorescent-activated cell sorting (FACS) with a BD FACSARIA™ IIIu to enrich the positive cell populations based on GFP expression. Sorted cells were further cultured to generate an appropriate stock maintained frozen in liquid nitrogen until use.

**Table 2:** CRISPR guide sequences.

Gene name	Guide name	Guide sequence (5’-3’ + PAM site)	Strand	Genome coordinate (GRCh38/hg38)
*IL32*	1076_crRNA_1	GCTTCTTCATGTCATCAGAG AGG	–	Chr16: 3, 067, 379-3, 067, 401
*IL32*	1076_crRNA_2	TGATGACATGAAGAAGCTGA AGG	+	Chr16: 3, 067, 385-3, 067, 407
*IL32*	1076_crRNA_3	GACAGTGGCGGCTTATTATG AGG	+	Chr16: 3, 068, 206-3, 068, 228

CRISPR, Clustered Regularly Interspaced Short Palindromic Repeats; IL32, interleukin 32; crRNA; crispr RNA; PAM, Protospacer Adjacent Motif; GRCh38, Genome Reference Consortium Human Build 38 Organism; Chr, chromosome; G, guanine; T, thymine; C, cytosine; A, adenine.

### Tracking of indels by decomposition analysis

HT-29 enriched cell populations from each electroporation conditions were pelleted prior genomic DNA extraction using MyTaq Extract PCR kit according to the manufacturer (Cat. no. BIO-21126, Bioline, London, United Kingdom). PCR amplification was performed using the high-fidelity enzyme Q5 (Cat. no. M0530L, N.E.B., Ipswich, Massachusetts, USA), using locus specific primers listed in **Table 3**. The PCR products obtained were sent for sequencing at the *Centre d’expertise et de service de Génome Québec* (CHU-Ste-Justine, Montréal, Canada). Sequence alignments were analyzed using the Snapgene software (Dotmatics, https://www.snapgene.com/) and cleavage activity was assessed using the TIDE software (Synthego, https://tide.nki.nl/).

**Table 3:** Primer sequences.

Gene name	Primer name	Primer sequence (5’-3’)	Strand	Genome coordinate (GRCh38/hg38)
*IL32*	1076_Fwd2	GTCTTAGGCTCCACAGGACACT	+	Chr16: 3, 068, 051-3, 068, 072
*IL32*	1076_Rv2	AAGAAAGAAAAGACAGGGCAGA	–	Chr16: 3, 068, 283-3, 068, 304

IL32, interleukin 32; Fwd, forward primer; Rv, reverse primer.

### Whole-genome RNA sequencing

Whole genome RNA sequencing and analysis were performed, as we reported (33). Briefly, total RNA was extracted from *CTL* and *IL32KO IEC* stimulated in the presence or the absence of hrTNF-α (10 ng/ml) for 18 hours, using the All Prep DNA/RNA/miRNA Universal Kit (#80224, Qiagen, MD, USA). The RNA sequencing was performed using the Illumina NovaSeq 6000 PE100-25M Reads Technology at the McGill University and Génome Québec Innovation Centre (Montréal, QC, Canada). Paired-end sequencing reads were pseudo-aligned to coding and non-coding transcripts from the Homo sapiens GRCh38 Ensembl release 108 reference transcriptome and quantified using Kallisto v0.50.1. Transcript-level counts were summarized to gene level using tximport. Genes with fewer than 1 count per million (CPM) in at least 2 samples were filtered out prior to normalization. Raw counts were transformed into log2-CPM values using the voom method from the limma Bioconductor R package (v3.64.1). Differential expression analysis was performed using limma with duplicate correlation to account for biological replicates. Differentially expressed genes (DEG) were identified based on adjusted p-values (adj. p < 0.05, Benjamini-Hochberg correction) and a fold-change cutoff of 1.3. Gene set variation analyses (GSVA) was performed using the GSVA Bioconductor package. Statistical analyses were performed using R v4.5.1. The entire RNA-seq dataset and the technical information requested by Minimum Information About a Microarray Experiment (MIAME) are available at the Gene Expression Omnibus (GEO) database under accession GSE320067 (https://www.ncbi.nlm.nih.gov/geo/query/acc.cgi?acc=GSE320067).

### Statistical analysis

Statistical analyses were conducted using the GraphPad Prism 8 software (Dotmatics). The nonparametric Friedman with Dunn’s multiple comparisons tests were applied to determine statistical significance between matched groups. P-values <0.05 were considered significant.

## Results

### Novel regulators of IL-32 expression in IEC

**Figure 1:**
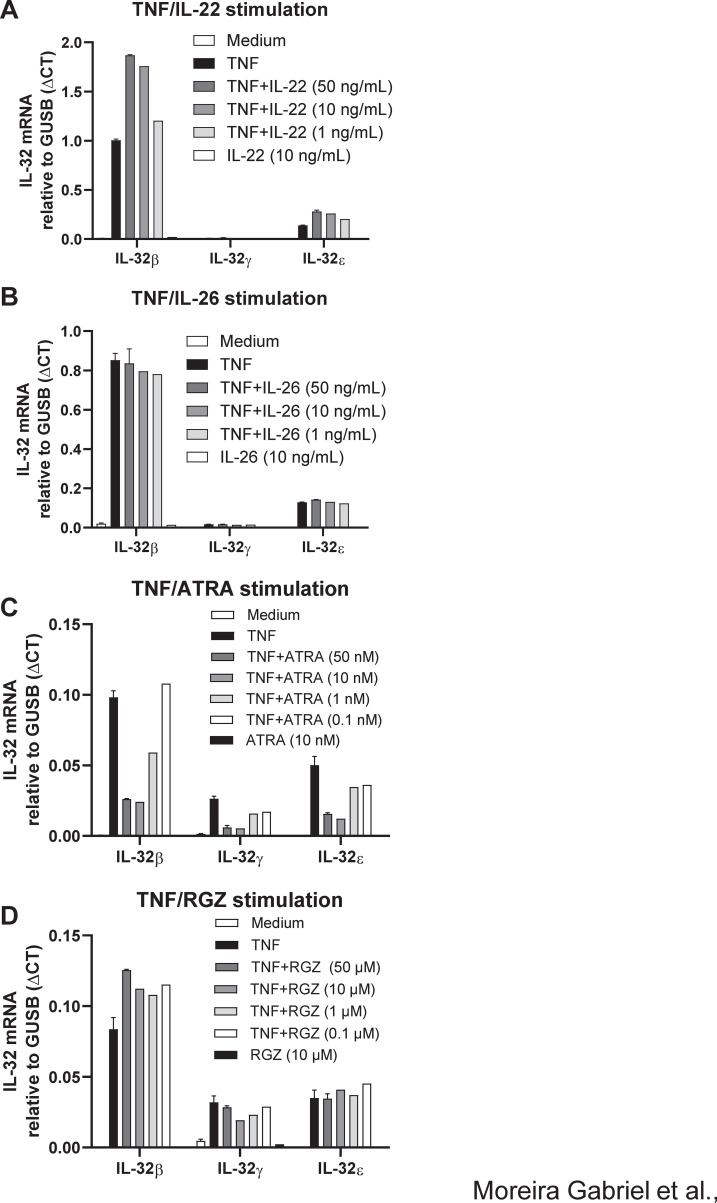
Identification of new modulators of IL- mRNA expression in IEC. HT-29 intestinal epithelial cells (IEC) at 70-80% confluence were exposed to different concentrations of human recombinant interleukin (IL)-22 and IL-26 (50, 10 and 1 ng/mL), as well as *all-trans* retinoic acid (ATRA) (50, 10, 1 and 0.1 nM) and rosiglitazone (RGZ) (50, 10, 1 and 0.1 µM), in the presence or the absence tumor necrosis factor (TNF)-α (10 ng/mL) for 18 hours. Cells were harvested, total RNA extracted and used to measure IL-32β/γ/ε mRNA expression by real-time RT-PCR. Results were normalized to *β-glucuronidase* (*GUSB)* mRNA expression, as housekeeping gene. RT-PCR reactions were performed in duplicates. Shown are results (mean ± SD) from one experiment representative of results generated with HT-29 IEC from 2 **(A, B)** and 4 **(C, D)** different passages, between passages 3 and 9.

In recent studies, we identified the Th17 hallmark cytokine IL-17A as a negative regulator of IL-32 expression in TNF-activated IEC (33, 41). To explore the antiviral features of IL-32 in IEC, we first sought to determine whether other Th17 cytokines, such as IL-22 and IL-26 (18), modulate IL-32 expression in HT-29 IEC. TNF activation was used as a positive control for IL-32 induction (33, 41, 51). Results in **Figure 1** demonstrate that, among IL-32 isoforms, IL-32β mRNA was expressed at the highest levels by IEC, while IL-32γ and IL-32ε mRNA were expressed at low/intermediate levels. In the presence of TNF, IL-22 increased IL-32β and IL-32ε but not IL-32γ mRNA expression, mainly at the highest IL-22 concentration of 50 ng/ml (**Figure 1A**). In contrast, IL-22 alone did not induce the expression of any IL-32 isoform tested (**Figure 1A**). Thus, the IL-22 action requires the activation with TNF of IEC, likely for upregulating IL-22 receptor expression. This is similar to IL-17A (33), whose receptors are not expressed constitutively on IEC, but rather are induced by activation with TNF (33, 52). Finally, IL-26 alone or together with TNF did not induce the expression of IL-32 isoforms on IEC (**Figure 1B**).

In addition to Th17-specific cytokines, we further aimed to identify pharmacological modulators of IL-32 expression. We focused on the RARA ligand *all trans* RA (ATRA) and the PPARγ ligand rosiglitazone (RGZ), two regulators of HIV-1 replication in CD4^+^ T-cells and macrophages (45–47).This choice was also justified by the *in silico* identification of putative RA responsive elements (RARE) (https://www.genecards.org/cgi-bin/carddisp.pl?gene=IL32) and PPARγ responsive elements (PPRE) (https://www.genecards.org/cgi-bin/carddisp.pl?gene=IL32) (53, 54) in the *IL32* gene promoter/enhancer, indicative of a potential direct regulation of *IL32* gene transcription by RARA and PPARγ. ATRA, at both 50 nM and 10 nM, decreased the expression of IL-32β/γ/ϵ mRNA in TNF-activated IEC (**Figure 1C**), while RGZ in combination with TNF slightly increased IL-32β mRNA expression (**Figure 1D**). Of note, ATRA and RGZ in the absence of TNF did not influence IL-32 mRNA expression in IEC (**Figures 1C, D**).

Thus, we identified IL-22, ATRA and RGZ as novel modulators of IL-32 β/γ/ϵ mRNA expression in TNF-activated IEC.

### Effect of IL-22, IL-26, ATRA and RGZ on HIV-1 outgrowth in IEC:T-cell co-cultures

**Figure 2:**
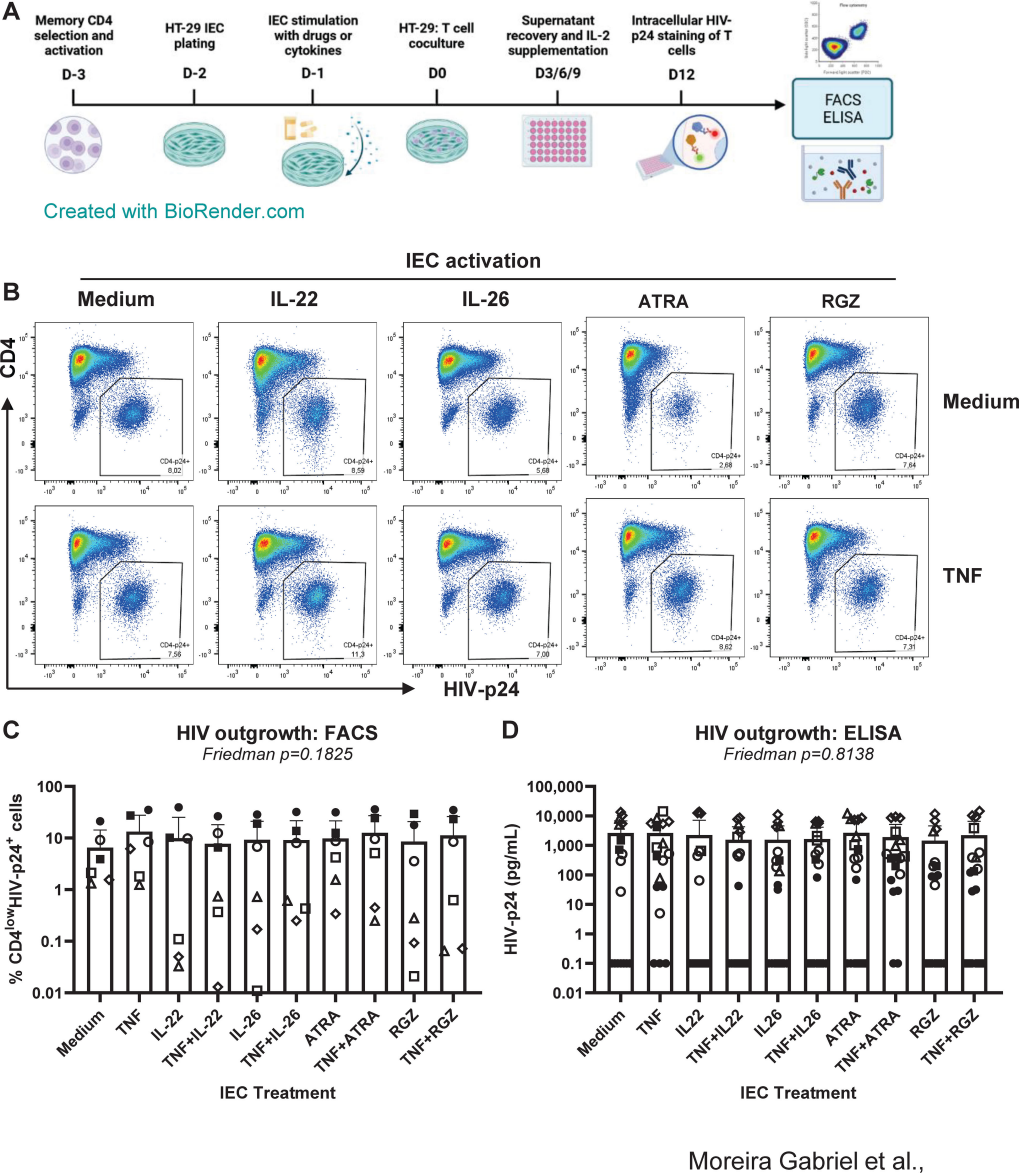
Effect of TNF and/or IL-22, IL-26, ATRA and RGZ on the ability of IEC to modulate HIV-1 outgrowth in CD4^+^ T-cells of PWH. **(A)** The flowchart outlines the experimental procedure for IEC:T-cell co-cultures. Briefly, CD4^+^ T-cells were isolated by negative section using magnetic beads from PBMCs of ART-treated PWH and activated with anti-CD3/CD28 Abs for 3 days. Concurrently, the HT-29 IEC (10^5^ cells/48-well plate) were cultured overnight until 70-80% confluence and then co-cultured with TCR-activated CD4^+^ T-cells (10^6^ cells/48-well plate). Co-cultures were maintained for 12 days in the presence of IL-2 (5 ng/ml). Media containing IL-2 was refreshed every 3 days. At day 12 post-co-culture, cells were harvested, pooled, and stained on the surface with CD3 and CD4 Abs and intracellularly with HIV-p24 Abs (**Supplementary Figure 1**). Supernatants collected were used to quantify soluble HIV-p24 protein by ELISA. **(B, C)** Shown are representative flow cytometry CD4/HIV-p24 dot plots **(B)** and statistical analysis of the frequencies of CD4^low^HIV-p24^+^ T-cells **(C)** when IEC stimulated or not with IL-22 (50 ng/ml), IL-26 (50 ng/ml), ATRA (10 nM), and RGZ (50 µM) in the presence/absence of TNF (10 ng/ml) were co-cultured with CD4^+^ T-cells of ART-treated PWH (n=6) for 12 days. **(D)** Shown are HIV-p24 levels measured by ELISA in cell culture supernatants harvested at day 12, with individual and median values shown. Friedman p-values and significant (p<0.05) Dunn’s post-test p-values are indicated on the graphs. Each symbol represents one participant. Experiments were conducted in triplicates. Identical wells were merged for FACS analysis of HIV-p24 expression **(C)**, while ELISA HIV-p24 quantification was performed for each replicate individually **(D)**.

Given the reported antiviral features of IL-32 (37, 38), we further aimed to explore the influence of IL-32 modulators on the ability of IEC to regulate viral outgrowth in CD4^+^ T-cells carrying HIV-1 reservoirs. To this aim, we performed an IEC-based VOA using a protocol we reported previously (33), where cytokine-activated IEC were co-cultured with CD3/CD28-activated CD4^+^ T-cells from ART-treated PWH (**Figure 2A**). Productively infected T-cells were identified by flow cytometry at day 12 post-culture, as cells with a CD4^low^HIV-p24^+^ phenotype (**Supplementary Figure 1**), consistent with previous studies (55, 56). To assess the effect of IEC co-culture on HIV outgrowth in CD4^+^ T-cells, the VOA was performed in the presence or the absence of IEC (**Supplementary Figure 2**). The % of CD4^low^HIV-p24^+^ T-cells was significantly higher in CD4^+^ T-cells cultured in the presence *versus* the absence of IEC (**Supplementary Figures 2A, B**), while levels of soluble HIV-p24 were similarly high in the two VOA conditions (**Supplementary Figure 2C**), indicative of an efficient HIV-1 outgrowth in the presence of IEC. Subsequent experiments were performed with IEC activated with IL-22, IL-26, ATRA or RGZ in the presence or the absence of TNF. Differences in the frequency of productively infected CD4^low^HIV-p24^+^ T-cells did not reach statistical significance, despite a tendency for decrease observed when IEC were exposed to IL-22, IL-26 and RGZ alone (**Figures 2B, C**). Similarly, no statistically significant differences were observed when soluble HIV-p24 levels were measured in the cell-culture supernatants (**Figure 2D**). In conclusion, these results reveal that the modulation of IL-32 expression by IL-22, RGZ and ATRA in TNF-activated IEC is not associated with proportional changes in HIV-1 outgrowth from CD4^+^ T-cells.

### CRISPR-Cas9 *IL32* gene editing in IEC

**Figure 3:**
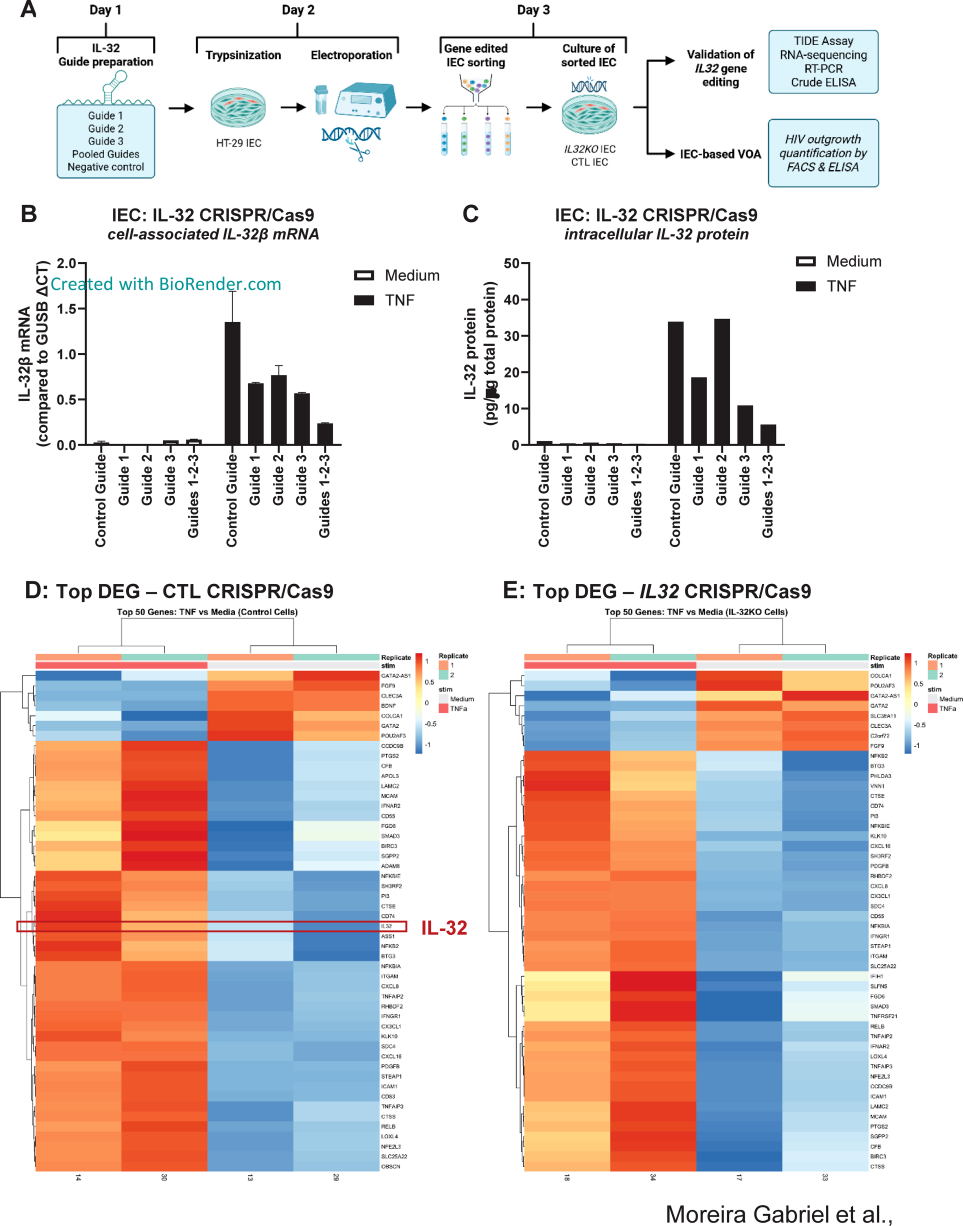
Impact of CRISPR-Cas9-mediated *IL32* gene editing on IEC transcriptional reprogramming upon TNF exposure. **(A)** Shown is the experimental flowchart of CRISPR-Cas9-mediated *IL32* gene knockout (*IL32*KO) in HT-29. Briefly, IEC were electroporated with 3 different guide RNA targeting the *IL32* gene individually or pooled together, and a negative guide RNA was used as control. Edited *IL32*KO and control (*CTL*) HT-29 cells were sorted based on ATTO 550 dye expression, cultured and used for gene editing validations and IEC-based VOA. **(B-E)** Briefly, *IL32KO* and CTL IEC were exposed or not to TNF (10 ng/ml) for 18 hours, harvested, and used for total RNA extraction or stored as dry pellets for crude ELISA. **(B, C)** The efficacy of *IL32* gene editing was further investigated by **(B)** RT-PCR quantification of IL-32β mRNA and **(C)** crude ELISA quantification of total intracellular IL-32 protein. **(D, E)** Genome-wide transcriptional profiling was performed using the Illumina RNA sequencing technology. Shown are differentially expressed genes (DEG) identified based on fold change (FC) expression in TNF *versus* Media conditions. Heat maps depict top 50 DEG in CTL **(D)** *versus IL32*KO **(E)** IEC in response to TNF activation, with upregulated transcripts in red and downregulated transcripts in blue. Indicated in the upregulation of IL-32 mRNA expression in CTL but not IL32KO IEC in response to TNF. Experiments were performed with *IL32KO* and CTL IEC from 2 **(B)**, 4 **(C)** or 2 **(D, E)** different passages. Shown are results (mean ± SD) from one representative experiment repeated twice **(B)**, results (mean ± SEM) from 4 different experiments, with each symbol representing one experiment **(C)**, as well as results from 2 different replicate experiments **(D-E)** performed with IEC from different passages, from passages 3-9.

To explore more deeply the functional role of IL-32 expression in IEC, we used the CRISPR/Cas9 gene editing technology to knockout (KO) the *IL32* gene in HT-29 cells (**Figure 3A**). Briefly, three different IL-32 guide RNAs, used individually or pooled, and a control guide RNA were inserted by electroporation in HT-29 IEC. Efficiently transfected cells were sorted by flow cytometry based on ATTO 550 dye expression and cultured to generate a stock of *IL32*KO and control IEC, subsequently used for the validation of CRISPR/Cas9-mediated *IL32* gene editing (**Figure 3A**). Results in **Supplementary Figure 3A** display the TIDE analysis of the *IL32* DNA sequences in *IL32*KO and control IEC. The editing of the *IL32* gene induced by insertion/deletions (indels) for cells electroporated with Guide 3 and pooled Guides 1-2–3 RNA is highlighted in red squares. Results in **Supplementary Figure 3B** indicate the editing efficacy of pooled Guide 1-2–3 RNA, as reflected by the presence of indels. Results in **Supplementary Figure 3C** depict the PCR amplification products used to confirm the guide cutting efficiencies (before sequencing). Finally, results in **Figures 3B, C** indicate a robust decrease in IL-32 mRNA (**Figure 3B**) and cell-associated IL-32 protein (**Figure 3C**) levels in *IL32*KO HT-29 cells, mainly in those transfected with the pooled Guides 1-2–3 RNA compared to control-transfected HT-29 cells, and this upon TNF-mediated activation. Given the high efficacy of CRISPR/Cas9-mediated *IL32* gene editing using the Pooled Guide 1-2–3 RNA, the subsequent experiments were performed using these cells (*IL32*KO), as well as the control IEC (CTL).

To evaluate the off-target effect of CRISPR/Cas9-mediated *IL32* gene editing in HT-29 cells, RNA extracted from *CTL* and *IL32KO* IEC, activated or not *via* TNF, served for genome-wide RNA sequencing using the Illumina technology. Results in **Supplementary Files 1**, **2** reveal that TNF efficiently modulated gene expression in both *CTL* and *IL32KO* IEC, with a total number of 1, 682 and 1, 562 DEG, respectively, identified based on adjusted p-values (p<0.05) and fold changes (FC, cut-off 1.3). Top 50 DEG included IL-32 mRNA in the case of TNF-activated *CTL* but not *IL32KO* IEC (**Figures 3D, E**), indicative of an effective *IL32* KO. Further, the transcriptional profiles were compared between *CTL* and *IL32KO* IEC under constitutive conditions or upon TNF activation. Differences in gene expression did not reach statistical significance with adjusted p-values (**Supplementary Files 3**, **4**). The analysis of DEG based on simple p-values (p<0.05) and FC (± 1.3) revealed 208 DEG upon TNF activation, with CX3CL1/Fractalkine transcripts expressed a lower levels in *IL32KO versus CTL* IEC (**Supplementary File 4**), indicative of a reduced inflammatory potential of *IL32KO* IEC.

These results point to efficient *IL32* KO and limited off-target effects of CRISPR/Cas9-mediated *IL32* gene editing in HT-29 IEC and reveal a minor impact of intrinsic IL-32 expression on the transcriptional reprogramming of IEC in response to TNF, pointing to a potential role of IL-32 in exacerbating inflammation in IEC.

### Effect of CRISPR-Cas9 *IL32* gene editing in IEC on HIV-1 outgrowth

**Figure 4:**
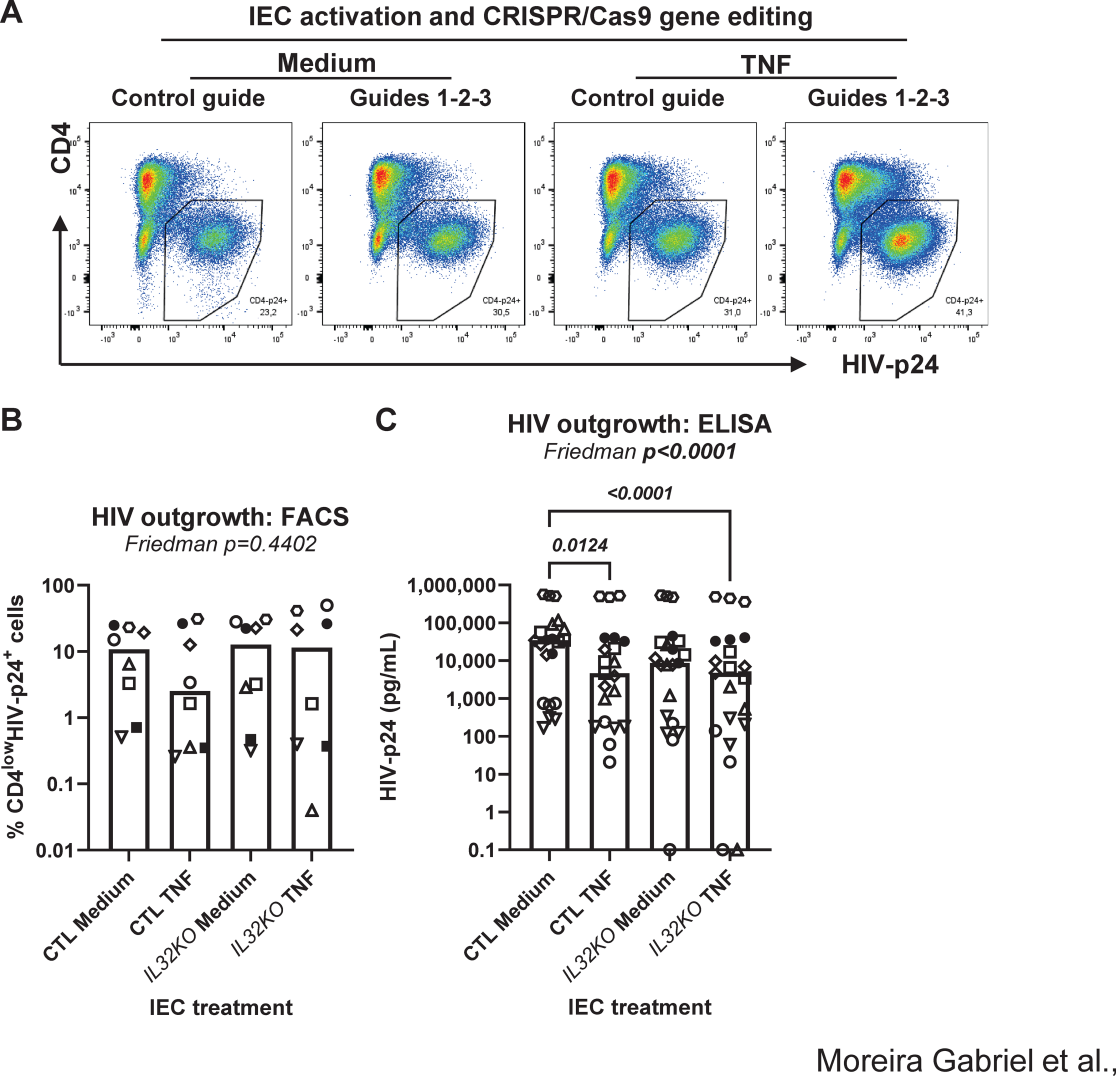
Effect of IL32 gene editing in IEC on the efficacy of viral outgrowth in CD4^+^ T-cells of ART-treated PWH. The CTL and *IL32KO* IEC were generated, activated or not with TNF (10 ng/ml) for 18 hours, as in **Figure 3A**, and co-cultured with TCR-activated CD4^+^ T-cells of ART-treated PWH (n=8), as in **Figure 2A**. Shown are representative flow cytometry dotplots of CD4/HIV-p24 expression **(A)** and statistical analysis of the frequencies of CD4^low^HIV-p24^+^ T-cells at day 12 post co-culture **(B)**, as well as the statistical analysis of HIV-p24 levels in cell-culture supernatants as measured by ELISA **(C)**. Friedman p-values and significant (p<0.05) Dunn’s post-test p-values are indicated on the graphs. Experiments were conducted in triplicates. Identical wells were merged for FACS analysis of HIV-p24 expression **(B)**, while ELISA HIV-p24 quantification was performed for each replicate individually **(C)**.

Finally, we explored differences in HIV-1 outgrowth when CD4^+^ T-cells of ART-treated PWH were co-cultured with *IL32*KO and *CTL* HT-29 IEC stimulated or not with TNF, with co-cultures performed as in **Figure 2A**. Flow cytometry analysis at day 12 post-co-culture revealed similar frequencies of productively infected CD4^low^HIV-p24^+^ T-cells when the IEC-based VOA was performed with *IL32*KO and CTL IEC (**Figures 4A, B**), with no differences between medium and TNF conditions (**Figure 4B**). The quantification of soluble HIV-p24 in cell culture supernatants collected at day 12 of co-culture revealed a significant decrease in HIV-1 replication when *CTL* IEC were exposed to TNF (Friedman p<0.0001; Dunn’s post-test p=0.0124), as well as a decrease between TNF-activated *IL32*KO and medium *CTL* IEC (**Figure 4C**; Dunn’s post-test p<0.0001). This slight decrease is consistent with the capacity of TNF to induce a type I IFN-mediated antiviral program in IEC (33). Nevertheless, the genetic manipulation of IL32 expression in IEC demonstrated no statistically significant impact on HIV-1 outgrowth under TNF-activated conditions (**Figure 4C**).

Altogether, these results demonstrate that IL-32 expression in TNF-activated IEC does not interfere with HIV-1 outgrowth in neighboring CD4^+^ T-cells of ART-treated PWH and that TNF exerts antiviral roles *via* mechanisms independent on IL-32.

## Discussion

In this study, we tested the hypothesis that inflamed IEC govern the transcriptional/translational activity of HIV-1 reservoirs in gut-infiltrating CD4^+^ T-cells of ART-treated PWH *via* mechanisms dependent on IL-32. This hypothesis was supported by the evidence in the literature documenting that IL-32 exerts antiviral functions against HIV-1 and other viruses (37, 38, 57, 58), as well as our studies demonstrating that IL-17A-activated IEC promote HIV-1 outgrowth in CD4^+^ T-cells of ART-treated PWH, while decreasing IL-32 expression in IEC (33, 41). Our study ***i)*** identified new positive (*i.e.*, IL-22, RGZ) and negative (*i.e.*, ATRA) modulators of IL-32 expression in IEC that acted exclusively in combination with TNF; ***ii)*** demonstrated that IEC exposure to IL-22, IL-26, or RGZ alone tended to decrease HIV-1 outgrowth in IEC:T-cell co-cultures, without an effect on intrinsic IL-32 expression; ***iii)*** and revealed that CRISPR/Cas9-mediated *IL32* gene editing did not affect the ability of IEC to modulate HIV-1 outgrowth in CD4^+^ T-cells of ART-treated PWH. Our identification of novel positive/negative regulators of IL-32 expression is highly relevant for new therapeutic interventions, given the documented implications of IL-32 in various HIV-associated co-morbidities, such as cardiovascular disease (CVD) (39, 40, 41 11136, 42, 59, 60). Our findings that IL-32 appears to have no antiviral effect in the context of the crosstalk between IEC and CD4^+^ T-cells, support the idea of a safe IL-32 targeting strategy to reduce the negative consequences of IL-32 overt expression in ART-treated PWH (39–41, 44).

The IL-32 cytokine family includes various isoforms that exert a wide variety of pro- and anti-inflammatory functions (61, 62), as well as antiviral effects against HIV-1 and other viruses (37, 38, 57, 58, 63). Our prior research provided a cartography of IL-32 isoform expression in sigmoid colon biopsies of ART-treated PWH *versus* uninfected controls and demonstrated that the overexpression of IL-32β coincided with the paucity of IL-17A mRNA (41), suggesting that the Th17 deficit underlie the IL-32 overexpression at mucosal level in PWH. Moreover, we demonstrated that exposure to TNF, upregulated IL-32β mRNA/protein expression in HT-29 IEC, with IL-17A decreasing IL-32 expression in these conditions *in vitro* (41). Furthermore, we demonstrated an increased HIV-1 outgrowth when CD4^+^ T-cells of ART-treated PWH were co-cultured with IL-17A-activated IEC (33). Whether IL-32 exerts antiviral effects in the context of the crosstalk between IEC and CD4^+^ T-cells remains unknown. In line with this possibility, IEC were documented to promote an antiviral state in neighboring CD4^+^ T-cells (64). Interestingly, Guo et al. reported that IEC produce antiviral factors for fighting HIV-1 and that the supernatant of IEC cultures reduce HIV-1 replication in macrophages (65). At the opposite, Gornalusse et al. discovered that primary endocervical epithelial cells induced HIV-1 reactivation of from latently infected T-cells *in vitro*, raising the possibility that epithelial cells could favor HIV-1 rebound *in vivo* (66). In this context, we first sought to identify new regulators of IL-32 expression in IEC that impact on HIV-1 reservoirs in CD4^+^ T-cells. We first focused on two other Th17-lineage cytokines, IL-22 and IL-26, documented to interact with epithelial cells during homeostasis and disease pathogenesis (67).

IL-22, produced by Th17/Th22-polarized CD4^+^ T-cells, interacts with IEC *via* binding on specific receptors expressed on the intestinal epithelial layer (67–69). During inflammation, IL-22 can exert dual roles, promoting tissue repair or driving inflammatory responses (68, 70, 71). Notably, reports have indicated that IL-22 exerts antiviral functions in the context of HIV-1 infection (72, 73). In contrast to IL-22, the IL-26 biological functions are less documented, mainly because its absence in rodents (67); however, its contribution to chronic intestinal inflammatory conditions is increasingly recognized (29, 74–76). IL-26, produced also by human Th17-polarized CD4^+^ T-cells, acts as a pro-inflammatory cytokine at epithelial barriers (29, 75–77). Interestingly, IL-26 has been shown to facilitate sensing of bacterial and self-DNA (29, 78, 79), suggesting a role in the innate immune defense. Elevated IL-26 levels following microbial translocation might represent an early frontline innate immune response (78, 80). Building on our previous findings that IL-17A-driven transcriptional reprogramming of IEC increased HIV-1 outgrowth by CD4^+^ T-cells of ART-treated PWH (33), we used here the same co-culture model to examine IEC activation by IL-22 and IL-26 in the presence/absence of TNF. Our results demonstrated that, in contrast to IL-17A (33), IL-22 synergized with TNF for an increased expression of IL-32 mRNA, while IL-26 did not affect IL-32 mRNA expression in IEC. Despite the capacity of IL-22 to increase IL-32β expression in TNF-activated IEC, there was no significant effect on HIV-1 outgrowth in IEC:T-cell co-cultures. In the absence of TNF, IL-22 or IL-26 alone did not induce IL-32 β/γ/ε expression in IEC, but slightly decreased HIV-1 outgrowth in a fraction of ART-treated PWH. These results point to IL-32-independent mediated effects of IL-22 and IL-26 on IEC functional crosstalk with CD4^+^ T-cells. In the context of HIV-1 infection, Kim et al. had shown the depletion of IL-22-producing T-cells from the GALT was associated with a compromised epithelial integrity and signs of microbial translocation, with exogenous IL-22 counteracting the HIV-induced damage to epithelial cells (73). The literature conveys that IL-22 possesses anti-HIV-1 and anti-inflammatory properties in different models (72, 81, 82). Imbalances in the function of IL-17/IL-22 in the blood and gut correlated with leaky gut and inflammation during chronic HIV-1 infection, despite ART treatment (83). Thus, IL-17A and IL-22 exert opposite effects on IL-32 expression, pointing to the essential role of these cytokines in maintaining mucosal homeostasis. The involvement of IL-26 in HIV-1 pathogenesis remains underexplored. Our group identified IL-26 expression in subsets of Th17-polarized CCR6^+^CCR4^+^ and Th1/Th17-polarized CCR6^+^CXCR3^+^ CD4^+^ T-cells that are highly permissive to HIV-1 infection (84, 85). Whether the Th17 deficit, well documented in PWH (18), is associated with a reduced expression of IL-26, thus creating an aberrant proviral environment, deserve further investigations.

Of particular interest, the promoter/enhancer region of IL-32 contains RA response elements (RARE) (https://www.genecards.org), suggesting a direct influence of RA on IL-32 transcription. RA is a vitamin A metabolite produced in part by mucosal DCs (86), which sustains gut integrity by controlling immune cell development, strengthening the intestinal barrier integrity, and fostering immune tolerance (87, 88). ATRA, the most abundant form of RA, engages with RA receptor alpha (RARA) and retinoid X receptor (RXR) to control specific gene transcription (86, 88–90). Among RA target genes, the gut-homing molecule integrin β7 was identified as a facilitator of HIV-1 entry (91, 92). The pro-viral effects of ATRA are supported by multiple other studies (86, 91, 93). Our group demonstrated that ATRA preferentially acts on CCR6^+^ CD4^+^ T-cells to increase their permissiveness to HIV-1 replication *in vitro* and HIV-1 outgrowth *ex vivo* (46, 94, 95), with similar proviral effects of ATRA being observed on macrophages (47). Of note, here we have found that ATRA significantly decreased IL-32 expression in TNF-activated IEC but had no effect on HIV-1 outgrowth in IEC:T-cell co-cultures, supporting the idea that IEC IL-32 expression does not play a role in the crosstalk with CD4^+^ T-cells.

In addition to RARE, the promoter/enhancer region of IL-32 also contains PPARγ responsive elements (PPRE) (https://www.genecards.org), pointing to PPARγ as a potential direct regulator of IL-32 transcription. PPARγ, in partnership with retinoic X receptor (RXR), binds on PPRE present in specific gene promoters (*e.g.*, genes involved in lipid/glucose metabolism), functioning either as a transcriptional repressor or an activator (96, 97). In addition to its metabolic roles, PPARγ is implicated in promoting the resolution of inflammation, mainly by inhibiting the activity of pro-inflammatory transcription factors, like NF-κB (96, 97). It is noteworthy that IL-32 promotes the aberrant accumulation of lipids in vascular walls and its overexpression is associated to atherosclerosis/cardiovascular disease (98, 99), particularly in PWH (40, 42, 59, 60, 100). Specifically, the IL-32α effects on lipid deposition and cholesterol efflux are modulated by PPARγ (54). In the present study, the PPARγ agonist RGZ slightly increased IL-32 expression in TNF-activated IEC, but exerted negligible effects on HIV-1 outgrowth in CD4^+^ T-cells of ART-treated PWH. At the opposite, RGZ alone had no effects on IL-32 expression in IEC but slightly decreased HIV-1 outgrowth in a fraction of ART-treated PWH. These antiviral effects are consistent with our reports identifying PPARγ as an intrinsic negative regulator of HIV-1 replication/outgrowth in Th17-polarized CD4^+^ T-cells (45, 84), as well as studies by other groups (101, 102).

To address the contribution of IL-32 to the crosstalk between IEC and CD4^+^ T-cells in the context of HIV-1 infection, we finally used the CRISPR/Cas9 gene editing technology to generate an *IL32*KO HT-29 cell line. As control, we used a HT-29 cell population exposed to the same gene editing manipulation, but in the absence of an *IL32-*targetting CreRNA guide. Our results demonstrated that HIV-1 outgrowth was similarly high CD4^+^ T-cells co-cultured with TNF-activated IEC regardless of their expression of IL-32, thus demonstrating that the intrinsic IL-32 expression in IEC has no role in this process.

The current study has limitations. First, we did not study the direct role of soluble IL-32 on HIV-1 outgrowth in CD4^+^ T-cells since IEC express IL-32 mRNA/protein intracellularly, but do not release soluble IL-32 (33, 41). However, soluble IL-32, produced by cells other than IEC, modulate T-cell functions (42, 43), including the reprogramming of CD4^+^ T-cells with a heart-homing tropism (40). Then, the mechanism by which IEC influence HIV-1 outgrowth in CD4^+^ T-cells beyond IL-32 was not addressed by this research. Novel investigations are required to identify molecular mechanisms mediating the crosstalk between IEC and CD4^+^ T-cells *in vitro* and *in situ*. As expected, there were donor-to-donor variations in HIV-1 outgrowth in the IEC:T-cell co-cultures. Other studies linked the size of replication-competent HIV-1 reservoirs to multiple clinical variables, including time since infection, time of ART initiation, ART regimen, age, and sex (1, 3). Our studies performed on CD4^+^ T-cells from ten male ART-treated PWH did not allow the stratification based on sex and other variables. Future studies are needed to determine if such differences influence the HIV-1 outgrowth in the context of the crosstalk between IEC and CD4^+^ T-cells. Additionally, we have not performed experiments on polarized IEC, nor on primary IEC. Nevertheless, studies on primary IEC previously performed by our group validated results obtained in HT-29 cells (33). Finally, although we excluded the direct contribution of IL-32 expressed by IEC in the modulation of HIV-1 outgrowth in CD4^+^ T-cells in this *in vitro* model of IEC-T-cell interaction, our studies were performed *in vitro* under static conditions and may underestimate the contribution of IL-32 to the complexity of inflammatory processes occurring at the gut epithelial barrier *in vivo*. Indeed, our RNA sequencing results revealed minor but not negligible differences in gene expression between TNF-activated *IL32KO* and *CTL* HT-29. Thus, under specific conditions *in vivo*, IL-32, by its capacity to induce TNF expression (61), may create a pro-inflammatory environment and attract more HIV-1 targets at mucosal barrier sites *in vivo*. Future studies on gut biopsies are needed to explore the role of IL-32 expression in the crosstalk between IEC and CD4^+^ T-cells at the intestinal barrier of ART-treated PWH *in situ*.

In conclusion, in this study we identified IL-22, ATRA and RGZ as novel regulators of IL-32 expression in TNF-inflamed IEC and demonstrated that intrinsic IL-32 expression by IEC has a negligible impact on HIV-1 outgrowth in CD4^+^ T-cells of ART-treated PWH (Graphical abstract). Our results are highly relevant in the context where IL-32 targeted therapeutic interventions are proposed as a strategy to reduce the risk of HIV-associated co-morbidities, such as CVD (34–36). Future studies *in vitro* and *ex vivo* are needed to identify molecular mechanisms by which IL-32, in the context of cytokines other than TNF, reprograms IEC in their ability to communicate with gut-infiltrating/resident CD4^+^ T-cells, with relevance for HIV-1 reservoir reactivation/latency as well as systemic inflammation during ART.

## Data Availability

The original contributions presented in the study are included in the article/**Supplementary Material**. Further inquiries can be directed to the corresponding author.

## Glossary

A: *adenine*
ART: antiretroviral therapy
ATRA: *All*-*trans* retinoic acid
Cas9: CRISPR-associated protein 9
Chr: *chromosome*
crRNA: *crispr RNA*
CRISPR: clustered regularly interspaced short palindromic repeats
CTL: control
DEG: differentially expressed genes
Fwd: *forward primer*
GALT: gut-associated lymphoid tissues
GEO: gene expression omnibus
GRCh38: *Genome Reference Consortium Human Build 38 Organism*
GSVA: gene set variation analysis
GUSB: *β-glucuronidase*
G: *guanine*
IEC: intestinal epithelial cells
IFN: interferon
IL: interleukin
ISG: interferon stimulated genes
HIV-1: human immunodeficiency virus type 1
KO: Knock-out
PAM: *Protospacer Adjacent Motif*
PPARγ: peroxisome proliferator activated receptor gamma
PPRE: PPARγ
PWH: people with HIV
RARA: retinoic acid responsive elements
Rv: *reverse primer*
RGZ: rosiglitazone
RXR: retinoid X receptor
SD: standard deviation
SEM: standard error of mean
TIDE: Tracking of Indels by Decomposition
Th: helper CD4^+^ T-cells
T: *thymine*
TNF: tumor necrosis factor
VOA: viral outgrowth assay.
