# Supplementary material for: Pharmacological and genetic modulation of IL-32 expression in intestinal epithelial cells does not impact HIV-1 outgrowth in co-cultured CD4+ T-cells
Source: Front Immunol. 2026 May 26;17:1769388. doi: 10.3389/fimmu.2026.1769388 (PMC13247358; doi:10.3389/fimmu.2026.1769388)
Supplement: Supplementary file 1 [file DataSheet1.pdf]

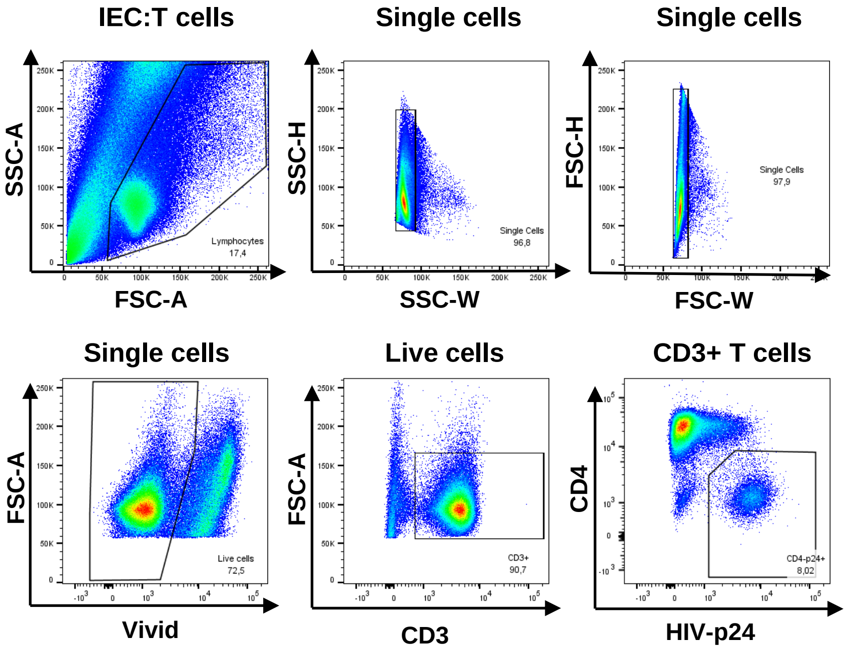


**Supplemental Figure 1 (related to Figures 1-3): Flow cytometry gating strategy for the identification of productively infected CD4$^{low}$HIV-p24$^{+}$ T-cells.** Memory CD4$^{+}$ T-cells from ART-treated PWH were stimulated via CD3/CD28 and cultured in the presence of HT-29 cells for 12 days. Cells were harvested and stained on the surface with CD3 and CD4 Abs and intracellularly with HIV-p24 Abs. Shown is the gating strategy illustrating the identification of productively infected CD3+ T-cells with a CD4$^{low}$HIV-p24$^{+}$ phenotype. The LIVE/DEAD Aqua Cell Stain dye was used for excluding dead cells from the analysis. Results are from one donor representative of results performed with cells from more than n=8 ART-treated PWH.

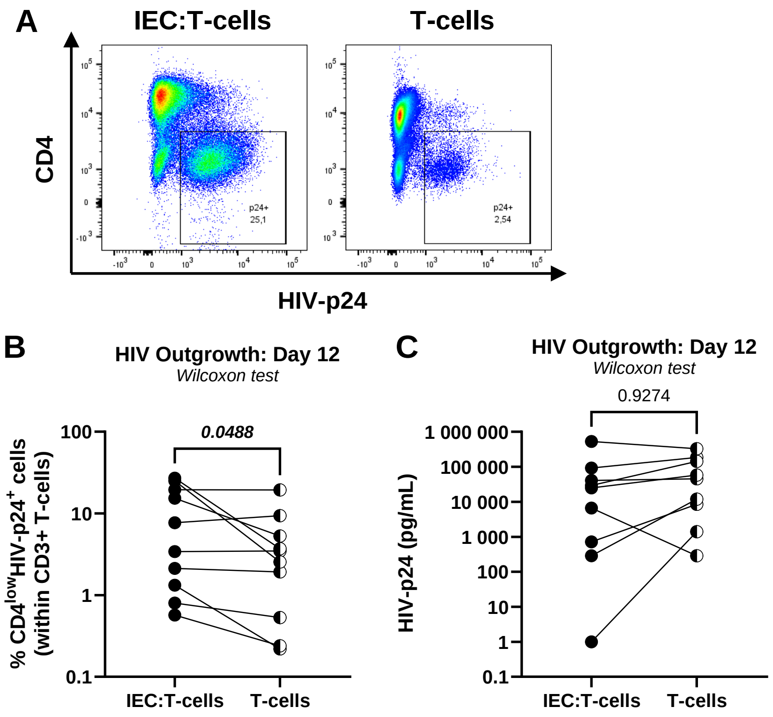


**Supplemental Figure 2: IEC sustain efficient HIV outgrowth from CD4$^+$ T-cells of ART-treated PWH.** The IEC:T-cell co-cultures were performed as depicted in Figure 2A.**(A)** Flow cytometry dot plots are from one representative donor and show the presence/frequency of productively infected T-cells (CD4$^{low}$HIV-p24$^+$) when T-cells were co-cultured with IEC (left, IEC:T-cells) and without IEC (right; T-cells). **(B-C)** Show are statistical analysis of HIV outgrowth in IEC:T-cell co-cultures *versus* single cultures of T-cells, as measured by flow cytometry in cells **(B)** and ELISA in cell-culture supernatants **(C)** harvested at day 12 post-co-culture. Wilcoxon p-values are indicated on the graphs. Experiments were conducted with T-cells of n=10 ART-treated PWH.

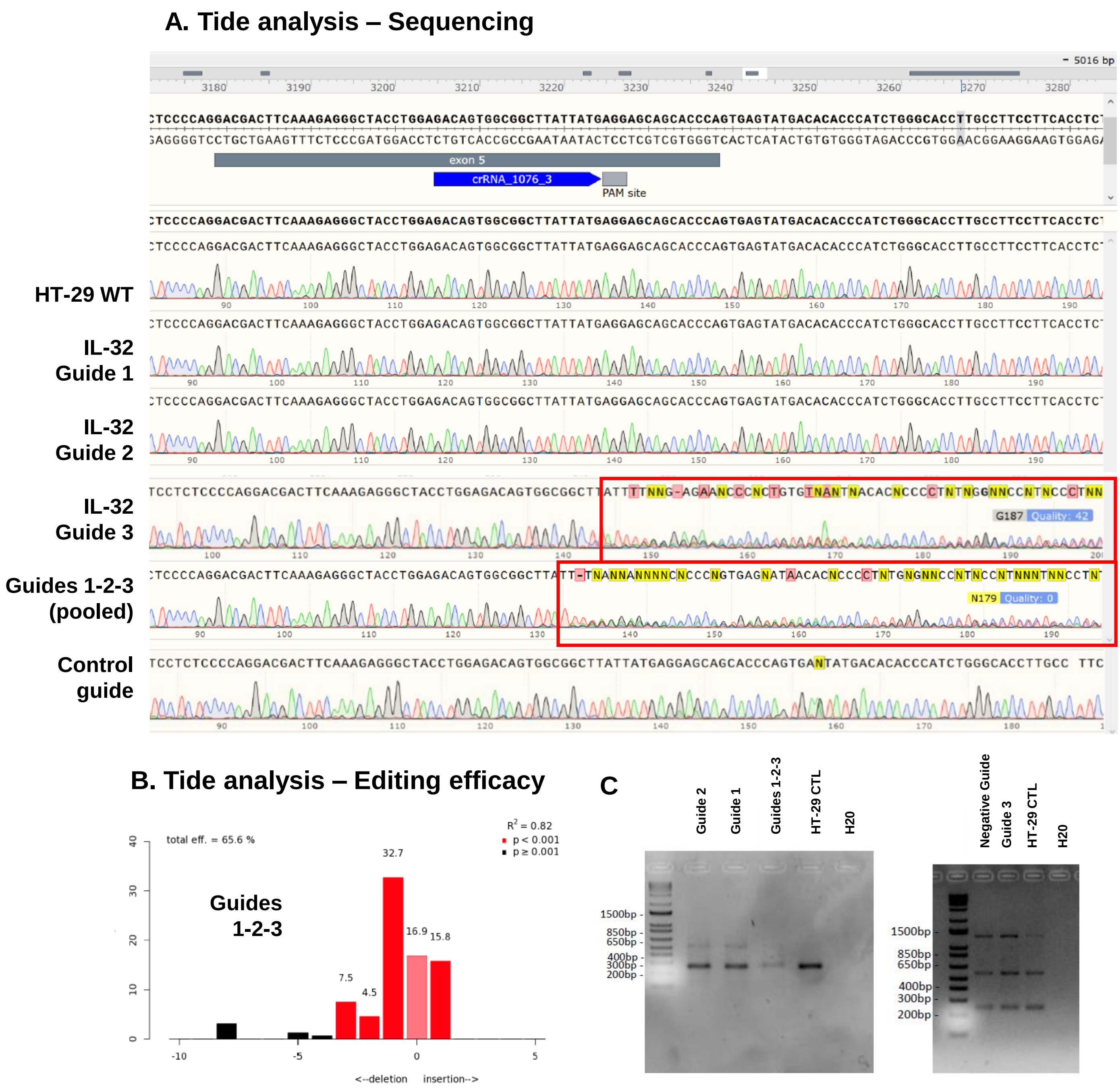


**Supplemental Figure 3 (related to Figure 3). CRISPR-Cas9-mediated IL-32 KO efficiency evaluation by TIDE, PCR and ELISA. (A)** The *IL32* gene sequence was analyzed in CRISPR-Cas9 *IL32* guide or control guide IEC populations using the Track of Indels by DEcomposition (TIDE). Indel editing in populations Guide 3 and Guides 1-2-3 is highlighted. **(B)** Editing efficacy of population Guides 1-2-3 is shown, indicating the presence of indels. **(C)** PCR amplification confirming guide cutting efficiencies (prior sequencing).
