# Supplementary material for: Pharmacological and genetic modulation of IL-32 expression in intestinal epithelial cells does not impact HIV-1 outgrowth in co-cultured CD4+ T-cells
Source: Front Immunol. 2026 May 26;17:1769388. doi: 10.3389/fimmu.2026.1769388 (PMC13247358; doi:10.3389/fimmu.2026.1769388)
Supplement: Supplementary file 2 [file DataSheet2.pdf]

CHUM

APPROUVÉ – CÉR CHUM

DATE : 5 juillet 2023
INITIALES : YP

FORMULAIRE D'INFORMATION ET DE CONSENTEMENT À L'INTENTION DU PARTICIPANT VIH POSITIF

**Titre de l'étude:**	Banque d'échantillons biologiques et de données cliniques y étant associées à des fins de recherche sur l'immunopathologie et la virologie du VIH-1
**Chercheuse responsable et Gestionnaire de la banque:**	Petronela Ancuta, Ph.D.<br>Professeure titulaire<br>Département de Microbiologie, infectiologie et immunologie<br>Faculté de Médecine, Université de Montréal<br>&<br>Responsable de laboratoire « Trafic cellulaire et pathogenèse du VIH »<br>Centre de Recherche du Centre Hospitalier de l'Université de Montréal (CRCHUM)
**Collaborateur Hors CHUM:**	Dr Jean-Pierre Routy, M.D. FRCPC<br>Directeur, cliniques VIH & Leucaphérèse<br>Service des maladies virales chroniques & Centre de médecine innovante<br>&<br>Directeur, Réseau SIDA et Maladies Infectieuses / FRQ-S<br>Institut de recherche du Centre Universitaire de Santé McGill (IR-CUSM)
**Organismes subventionnaires:**	Instituts de recherche en santé du Canada (IRSC)<br>Consortium canadien de recherche sur la guérison du VIH (CanCURE)
**No de projet au CHUM:**	22.267

PRÉAMBULE

Nous sollicitons votre participation à la mise en banque d'échantillons biologiques et de données cliniques y étant associées parce que vous vivez avec une maladie infectieuse (virus de l'immunodéficience humaine - VIH).

Nous vous invitons à poser toutes les questions que vous jugerez utiles au collaborateur du prélèvement et de la mise en banque ou aux membres de son équipe du CUSM et à leur demander de vous expliquer tous les éléments qui ne sont pas clairs. Si vous décidez de participer au prélèvement et à la mise en banque, vous devrez signer ce formulaire, dont une copie vous sera remise.

OBJECTIFS DE LA BANQUE D'ÉCHANTILLONS BIOLOGIQUES ET DE DONNÉES CLINIQUES Y ETANT ASSOCIÉES

Le but de ce prélèvement et de la mise en banque est de permettre aux chercheurs d'emmagasiner des échantillons biologiques, et leurs données cliniques y étant associées afin de mener des recherches dans le domaine de l'immunopathologie (science qui analyse les éléments liés au système immunitaire dans les échantillons biologiques) et de la virologie (science qui étudie les virus, agents infectieux de très petites tailles responsables de maladies) chez l'humain.

BUTS DE LA RECHERCHE EFFECTUÉE AVEC LES ÉCHANTILLONS MIS EN BANQUE

Le VIH est la cause du syndrome d'immunodéficience acquise (SIDA), qui entraîne une détérioration progressive des défenses de l'organisme contre les infections. Les globules blancs, en particulier les lymphocytes, sont responsables de coordonner les mécanismes de défense. Le VIH s'attaque précisément à un sous-groupe de lymphocytes, les lymphocytes T CD4+, et peut demeurer dans ces cellules malgré la prise de médicaments anti-VIH. Nous avons l'intention de mener ce projet de mise en banque pour mieux comprendre le processus de réduction du nombre de lymphocytes T CD4+ ainsi que les mécanismes responsables de la persistance du VIH durant le traitement de l'infection.

NATURE DE LA PARTICIPATION DEMANDÉE

En acceptant de participer au prélèvement et à la mise en banque et après avoir signé le présent formulaire d'information et de consentement, vous autorisez l'utilisation aux fins de recherche décrites au présent formulaire, de vos échantillons biologiques mis en banque et des données cliniques y étant associées provenant de votre dossier médical.

- **Mise en banque d'échantillons biologiques prélevés aux fins exclusives de la recherche**
  Vous êtes invité à faire don de vos globules blancs (y compris de vos lymphocytes) au moyen d'une technique de séparation cellulaire à flux continu. L'isolation des globules blancs des autres composants du sang est appelée leucaphérèse et nécessite l'utilisation d'un appareil capable d'extraire ces cellules. L'intervention dure environ trois (3) ou quatre (4) heures avec l'appareil de marque Optia. Toutefois, si vous décidez de faire don de vos globules blancs plus d'une fois, vous pourrez le faire un mois après la dernière leucaphérèse.

- **Mise en banque d'informations cliniques y étant associées**
  Les informations de votre dossier médical associées aux échantillons biologiques seront aussi mises en banque, car elles sont indispensables pour l'analyse des résultats de recherche. Ces informations incluent votre âge, votre sexe, votre groupe ethnique, votre diagnostic, votre état de santé actuel, les résultats de laboratoires, l'existence d'antécédents médicaux personnels et familiaux.

- **Prise de sang** :
  Une brève entrevue sera menée afin de connaître votre état de santé. Avant la leucaphérèse, des analyses sanguines seront réalisées (20 ml = 4 cuillères à thé de sang seront prélevés), telles qu'une sérologie pour déceler la présence des hépatites B et C, d'un cytomégalovirus, d'une syphilis et des HTLV-1 et 2. Si vous en donnez l'autorisation, tout résultat positif à l'un de ces tests vous sera communiqué, et vous serez référé à un spécialiste pour un suivi. Certains résultats d'analyse tels les hépatites peuvent devoir être signalés aux autorités gouvernementales, indépendamment de votre désir d'être recontacté ou non pour vos résultats.

  Oui____ Non____ J'accepte que l'on communique avec moi si j'obtiens un résultat positif à l'un de ces tests.

- **Leucaphérèse :**
  La leucaphérèse est réalisée au moyen d'un appareil automatique capable de recueillir les globules blancs par centrifugation à flux continu. Le sang est extrait d'une veine, puis passe dans une centrifugeuse, où les globules blancs sont retenus ; le reste du sang vous est ensuite restitué par une autre veine.

CONSERVATION DES ÉCHANTILLONS BIOLOGIQUES ET DES DONNÉES CLINIQUES Y ETANT ASSOCIÉES

Le produit de la leucaphérèse sera transporté au laboratoire de la Pr Petronela Ancuta, Ph.D., au Centre de recherche du CHUM, dans un colis réglementaire, par un service de transport accrédité et reconnu par Santé Canada et le CUSM et le CHUM. Les cellules seront alors isolées au moyen des techniques standards et pourront être utilisées fraîches (pour une cytométrie en flux ou pour l'étude des réservoirs du VIH), ou encore être conservées dans de l'azote liquide pour des recherches dans le domaine de l'immunopathologie et de la virologie chez l'humain.

AVANTAGES

Vous ne retirerez aucun avantage personnel direct de votre participation à la mise en banque et aux projets de recherche qui seront effectués à partir de vos échantillons biologiques et des données cliniques y étant associées. Toutefois, les recherches effectuées sur ces échantillons pourraient permettre aux chercheurs d'en apprendre davantage sur l'immunopathologie et la virologie chez l'humain. Cette information pourrait aider à l'avenir des patient(e)s atteint(e)s d'une maladie infectieuse qui pourraient participer à un projet de mise en banque semblable.

RISQUES

Lors des prises de sang, vous pourriez ressentir une faiblesse, un évanouissement ou présenter une légère douleur, une ecchymose (bleu), une irritation ou une rougeur au point de ponction. Dans de rares cas, une infection peut survenir.

Durant la leucaphérèse, il est possible que les bras deviennent fatigués ou engourdis en raison de leur immobilité. Les fourmillements ressentis au niveau de la bouche ou des doigts, le goût ou l'odeur inhabituelle et l'inconfort ou les spasmes musculaires associés à la diminution de la calcémie causée par l'anticoagulant pourront être soulagés par la prise d'un comprimé de calcium. Les phlébotomistes sont tous formés pour les prélèvements sanguins et possèdent donc, l'expérience nécessaire dans le domaine. Tous les efforts seront déployés pour réduire au minimum les réactions indésirables susceptibles de survenir. Les participants feront l'objet d'une étroite surveillance tout au long de la leucaphérèse afin de déceler la présence d'un effet secondaire et, si nécessaire, l'intervention sera ralentie, voire arrêtée avant la fin. La leucaphérèse est jugée sécuritaire chez les participants infectés par le VIH

- **Risque lié au bris de confidentialité :**

  Il existe un risque lié à un possible bris de confidentialité concernant vos informations personnelles et vos dossiers médicaux qui pourraient se traduire par une atteinte à votre vie privée. Ce risque est cependant minime. Tous les efforts seront déployés pour protéger votre vie privée et assurer votre confidentialité, tel que décrit dans la section «Confidentialité».

CONFIDENTIALITÉ

Tous les renseignements vous concernant demeureront strictement confidentiels dans les limites prévues par la loi.

Afin de préserver votre identité et la confidentialité des renseignements, vos échantillons biologiques et vos informations cliniques y étant associées seront identifiés par un numéro de code. La clé du code reliant votre nom à votre dossier de recherche et vos échantillons biologiques seront conservés par la gestionnaire de la banque. Par conséquent, seul la gestionnaire de la banque ou un membre désigné de son personnel pourra faire le lien entre les échantillons biologiques et vous-même.

Toutes les données vous concernant seront informatisées, protégées elles aussi par un accès sécurisé et accessible uniquement aux membres autorisés de la banque. L'accessibilité à ces données sera strictement contrôlée par la gestionnaire de la banque qui délivrera les autorisations d'accès.

Les informations vous concernant et découlant d'études faites avec vos échantillons biologiques serviront à des fins strictement scientifiques. Les résultats pourront être utilisés pour des conférences ou pour rédiger des articles scientifiques. Par contre, il ne sera pas possible de vous identifier.

On remettra des échantillons biologiques et les données cliniques y étant associées seulement à des chercheurs dont la responsable de la banque aura approuvé les propositions de recherche et qui sont liés par une entente de non-divulgation.

De plus, la responsable de la banque fournira les échantillons biologiques et les données cliniques pertinentes aux chercheurs qui en feront la demande uniquement si leur projet de mise en banque a été évalué et approuvé par le Comité d'éthique de la recherche de leur établissement.

L'accès aux échantillons biologiques codés se fera selon des modalités précises, énoncées dans le cadre de gestion de la banque.

Les rapports portant sur toute recherche effectuée au moyen de vos échantillons biologiques et les données cliniques y étant associées ne seront communiqués ni à votre médecin ni à vous, puisque les recherches réalisées sur vos échantillons biologiques et les données cliniques y étant associées n'auront pour vous aucune portée diagnostique ou thérapeutique. Ces rapports ne seront pas non plus versés dans votre dossier médical.

EN CAS DE PRÉJUDICE

Si vous deviez subir quelque préjudice que ce soit par suite de toute procédure reliée à votre participation à la banque à des fins de recherche sur l'immunopathologie et la virologie chez l'humain, vous recevrez tous les soins et services requis par votre état de santé.

En acceptant de participer à cette banque, vous ne renoncez à aucun de vos droits et vous ne libérez pas les chercheurs, le commanditaire, l'organisme subventionnaire et l'établissement de leur responsabilité civile et professionnelle.

PARTICIPATION VOLONTAIRE ET POSSIBILITÉ DE RETRAIT

Votre participation à la mise en banque des échantillons biologiques et des données cliniques y étant associées est tout à fait volontaire. Vous pouvez donc décider de ne pas participer à la banque sans que cela n'affecte la qualité de vos soins actuels ou futurs.

Si vous décidez de ne plus participer à la mise en banque et ne voulez plus qu'on utilise vos échantillons biologiques de la banque des échantillons biologiques et des données cliniques y étant associées à des fins de recherche sur l'immunopathologie et la virologie chez l'humain, vous devez en aviser le collaborateur et médecin-clinicien responsable qui transmettra la demande à la gestionnaire de la banque qui veillera à ce qu'on les détruise. Toutefois, si on a déjà soumis vos échantillons à des tests, ou si on a retiré le code originellement apposé sur votre échantillon, s'ils ont été anonymisés, si on les a inclus dans une analyse ou fait mention de résultats d'analyses dans une publication, il ne sera pas possible de les retirer.

POSSIBILITÉ DE COMMERCIALISATION

Vos échantillons biologiques serviront uniquement à des fins de recherche et ne seront pas vendus. Il se peut que la recherche utilisant vos échantillons contribue à la mise au point de nouveaux produits dans le futur qui pourraient être vendus. Cependant, vous ne pourrez en retirer aucun avantage financier.

COMPENSATION OU REMBOURSEMENT POUR LA PARTICIPATION

Vous ne recevrez aucune compensation financière pour votre participation à cette banque. Cependant vous recevrez un montant de 160.00$ en guise de compensation pour certains frais, encourus en raison de votre participation à la banque.

COMMUNICATION DES RÉSULTATS

Ni vous ni aucun membre de votre famille n'aurez accès à l'information dérivée de vos échantillons de globules blancs. Aucun renseignement relatif à vos échantillons biologiques ne vous sera transmis. Sauf si requis par un tribunal, aucun tiers, comme un employeur ou une compagnie d'assurance, ne recevra de l'information issue de vos échantillons dans le cadre du projet de mise en banque. Aucun résultat de recherche ne permettra de vous identifier.

PERSONNES-RESSOURCES

Si vous avez des questions au sujet de la mise en banque de vos échantillons biologiques et des données cliniques y étant associées à des fins de recherche sur l'immunopathologie et la virologie chez l'humain, veuillez contacter Dr Jean-Pierre Routy, collaborateur, médecin-clinicien responsable, le jour au 514-843-1558, ou, la coordonatrice de recherche, Mme Soumia Khalfi entre 8h00 et 16h00, au 514-890-8000*,* poste 31246.

Pour toute question concernant vos droits en tant que sujet participant à ce projet de mise en banque ou si vous avez des plaintes ou des commentaires à formuler vous pouvez communiquer avec le commissaire aux plaintes et à la qualité des services du CHUM, au 514-890-8484 ou de santé McGill au 514-934-1934, poste: 35655.

CONSENTEMENT

Je déclare avoir lu le présent formulaire de consentement (ou on me l'a lu), particulièrement quant à la nature de ma participation à la mise en banque et à l'étendue des risques qui en découlent. Je reconnais qu'on m'a expliqué la mise en banque, qu'on a répondu à toutes mes questions et qu'on m'a laissé le temps voulu pour prendre une décision.

Ces énoncés correspondent à une mise en banque associée avec un projet de recherche.

____

Nom (en lettres moulée)

SIGNATURE DE LA PERSONNE QUI A OBTENU LE CONSENTEMENT - CUSM

J'ai expliqué au (à la) participant(e) à la recherche les termes du présent formulaire d'information et de consentement et j'ai répondu aux questions qu'il (elle) m'a posé.

____

Nom (en lettres moulées) | Signature de la personne chargée de la discussion sur le consentement | Date

ENGAGEMENT DU RESPONSABLE DE LA BANQUE - CRCHUM

Je certifie qu'on a expliqué au/à la participant(e) le présent formulaire d'information et de consentement pour la banque, que l'on a répondu aux questions qu'il/elle avait.

Je m'engage, avec l'équipe de recherche, à respecter ce qui a été convenu au formulaire d'information et de consentement et à en remettre une copie signée et datée au/à la participant(e).

____

Nom (en lettres moulées) | Signature de la chercheuse responsable | Date

SIGNATURE D'UN TÉMOIN – CUSM OUI □ NON □

La signature d'un témoin est requise pour les raisons suivantes :

- ☐ Difficulté ou incapacité à lire - La personne (témoin impartial) qui appose sa signature ci-dessous atteste qu'on a lu le formulaire de consentement et qu'on a expliqué précisément le prélèvement et la mise en banque des échantillons biologiques et de données cliniques associées à des fins de recherche au (à la) participant(e), qui semble l'avoir compris(se).
- ☐ Incompréhension de la langue du formulaire de consentement - La personne qui appose sa signature ci-dessous a fait fonction d'interprète pour le ou la participant(e) au cours du processus visant à obtenir le consentement.

____

Nom (en lettres moulées) | Signature du témoin | Date

APPROBATION PAR LE COMITÉ D'ÉTHIQUE DE LA RECHERCHE

Le comité d'éthique de la recherche du CHUM a approuvé cette mise en banque d'échantillons biologiques et de données cliniques associées à des fins de recherche et en assurera le suivi pour les établissements du réseau de la santé et des services sociaux du Québec participants.
